# The effect of probiotics, prebiotics and synbiotics on gut microbial community profile in overweight and obese Latin American and Caribbean populations: a systematic review of human trials

**DOI:** 10.1017/gmb.2024.12

**Published:** 2025-01-17

**Authors:** Manahil M. Bineid, Litai Liu, Eduard F. Ventura, Sakshi Bansal, Katherine Curi-Quinto, Juana del Valle-Mendoza, Gemma E. Walton, Karani Santhanakrishnan Vimaleswaran

**Affiliations:** 1 Hugh Sinclair Unit of Human Nutrition, Department of Food and Nutritional Sciences, University of Reading, Reading,UK; 2 Department of Biotechnology, Institute of Agrochemistry and Food Technology, Spanish National Research Council (IATA-CSIC), Valencia, Spain; 3 Instituto de Investigación Nutricional, Lima, Peru; 4 Escuela de Nutrición de la Facultad de Ciencias de la Salud, Universidad Peruana de Ciencias Aplicadas, Lima, Peru; 5 Escuela de Medicina, Centro de Investigación e Innovación de la Facultad de Ciencias de la Salud, Universidad Peruana de Ciencias Aplicadas, Lima, Peru; 6 Food Microbial Sciences Unit, Department of Food and Nutritional Sciences, University of Reading, Whiteknights, Reading, UK; 7 The Institute for Food, Nutrition, and Health (IFNH), University of Reading, Reading, UK

**Keywords:** probiotics, prebiotics, synbiotics, gut microbiota, obesity, LACP

## Abstract

Oral supplementation with probiotics, prebiotics, and synbiotics is a novel potential complementary therapy for addressing overweight and obesity through gut microbiota modulation. This systematic review provides a comprehensive summary of the existing evidence to guide future research. Literature searches were conducted in four databases to identify human trials published until May 2024 that examined the impact of probiotic, prebiotic, or synbiotic interventions on faecal microbiota composition changes in overweight and obese participants from Latin American and Caribbean populations (LACPs). Of the 13,090 identified records, five randomised controlled trials (RCTs) from Brazil, Mexico, and Chile met the inclusion criteria for this review. The included RCTs evaluated different forms of therapies over short-term interventions (6 or 8 weeks), with sample sizes ranging from 21 to 39 participants across the studies. Variations in the reported outcomes were observed due to differences in supplement formulation, dosage, population characteristics, and methodological heterogeneity. The findings indicate that the available data are inadequate to establish definitive conclusions regarding the impact of biotic treatments on gut microbiota profiles in LACP. Further research with larger sample sizes and precise microbiota analysis is required to elucidate the implications of dietary interventions on gut microbiota in obesity and related disorders.

## Introduction

Obesity and overweight are major public health issues in the Latin American and Caribbean populations (LACPs). Obesity affects nearly a quarter of adults in the region, exceeding the global rate of 13.1% (FAO et al., 2021; The Lancet Regional Health-Americas, 2023). Between 2000 and 2016, the prevalence of adult obesity surged across all subregions, increasing by 9.5% in the Caribbean, 8.2% in Mesoamerica, and 7.2% in South America (Miranda et al., 2020; The Lancet Regional Health-Americas, 2023). The non-English-speaking Caribbean subregion experienced the fastest increase (Miranda et al., 2020). This alarming rise in obesity prevalence is primarily attributed to increasingly sedentary lifestyles and a significant shift in dietary patterns towards higher consumption of processed and energy-dense foods (FAO & PAHO, 2017; Popkin & Reardon, 2018; Webber et al., 2012). Diet is widely acknowledged as a pivotal determinant influencing the composition and diversity of the gut microbiome. Emerging evidence indicates a potential relationship between increasing obesity rates and changes in gut microbiota within the population (Magne et al., 2016). The intestinal microbial community contributes to the host’s metabolism and energy homeostasis, engaging in various activities such as short-chain fatty acid (SCFA) production (Clarke et al., 2014). Alterations in the microbial profile composition have been proposed as a key environmental driver of obesity (Gomes et al., 2018; Tagliabue and Elli, 2013). Differences in gut microbiota composition between obese and lean individuals have been observed, particularly concerning the relative abundances of three phyla: Bacteroidetes, Firmicutes, and Actinobacteria (Boroni Moreira et al., 2012; Chakraborti, 2015; Ley et al., 2006; Tehrani et al., 2012). An imbalance in gut microbiota composition is linked to obesity through several mechanisms, including increased energy extraction from the diet, altered fatty acid metabolism, and changes in gut peptide secretion (Musso et al., 2010). An altered gut microbial profile may further disrupt intestinal barriers, activate inflammatory pathways, and promote insulin resistance (Gomes et al., 2018). However, many of these proposed mechanisms whereby gut microbiota imbalance induces obesity have originated from animal models (Romieu et al., 2017; Tagliabue and Elli, 2013). Nonetheless, the intestinal microbiota is recognised as a novel factor that regulates body weight and the onset of chronic metabolic diseases owing to its involvement in the host’s physiological and immunological functions (Boroni Moreira et al., 2012).

Recently, modulating gut microbiota through external means has emerged as a new focus in obesity treatment (Crovesy et al., 2021; Dahiya et al., 2017; Ley et al., 2006). Among the proposed strategies, dietary applications of probiotics, prebiotics, and synbiotics have shown promise (Dahiya et al., 2017; Marchesi et al., 2016). Probiotics are defined as ‘live microorganisms that, when administered in adequate amounts, confer a health benefit on the host’ (Hill et al., 2014), while prebiotics are “substrates that are selectively utilised by host microorganisms, conferring a health benefit” (Gibson et al., 2017). Synbiotics are nutritional supplements that combine probiotics and prebiotics. According to the International Scientific Association for Probiotics and Prebiotics, synbiotics are a mixture comprising live microorganisms and substrate(s) selectively utilised by host microorganisms that confer a health benefit on the host (Swanson et al., 2020). Research demonstrated that biotic interventions positively impact weight loss, lipid profile, and glycaemic control in individuals with obesity and metabolic syndrome (Borgeraas et al., 2018; Crovesy et al., 2021; Dror et al., 2017; Hadi et al., 2019b; 2020a; 2021; Zhang et al., 2016). Additionally, it directly affects gut microbiota composition by boosting the growth of particular beneficial microorganisms (Clarke et al., 2014; Dahiya et al., 2017; Dror et al., 2017; Macfarlane et al., 2006). Prebiotic supplementation with dietary inulin-type fructans in women with obesity led to changes in the populations of Firmicutes, Actinobacteria, and Bacteroidetes and promoted the growth of several beneficial *Bifidobacterium* species (Dewulf et al., 2013; Salazar et al., 2015). Studies have shown varying results regarding the impact of probiotic interventions on gut microbial community (Lahtinen et al., 2012; Larsen et al., 2011. 2013; Plaza-Diaz et al., 2015). While probiotics may not always cause significant changes in the microbial population, they still modify gut microbiota through other mechanisms, such as metabolite production and microbial activity modulation (Olvera-Rosales et al., 2021; Sanchez et al., 2017). Additionally, the synergistic action of probiotics and prebiotics in synbiotic treatment offers combined benefits to host health (Patel and DuPont, 2015). For instance, administering the probiotic *Lactobacillus salivarius* Ls-33 to adolescents with obesity resulted in a significant increase in the ratio of the *Bacteroides-Prevotella-Porphyromonas* group to Firmicutes-belonging bacteria. However, this treatment did not affect obesity or related parameters, such as faecal SCFA concentration (Larsen et al., 2013). A follow-up study evaluated the effect of the probiotic *L. salivarius* alone and in combination with the prebiotic fructooligosaccharide (FOS) in healthy young participants (Rajkumar et al., 2015). Although both treatment groups showed positive changes, the synbiotic group (*L. salivarius* + FOS) exhibited a significantly greater decrease in total cholesterol, low-density lipoproteins cholesterol, and inflammatory markers compared with the other group. The synbiotic group demonstrated more pronounced alterations in gut microbiota populations, characterised by increased faecal counts of lactobacilli and a reduction in *Escherichia coli* and coliforms, suggesting that the synbiotic mixture may offer enhanced efficacy in addressing obesity by modulating key factors.

Recent evidence affirms that incorporating probiotics, prebiotics, and synbiotics into dietary interventions could serve as an innovative therapeutic strategy for modulating gut microbiota, offering potential for obesity treatment (Zsálig et al., 2023). Considering the diverse geographic locations and socioeconomic contexts of LACPs and the considerable variation in dietary and lifestyle habits (Magne et al., 2016), better comprehension of the link between diet, nutritional components, gut microbiota and obesity is needed. This highlights the importance of probiotics, prebiotics, and synbiotics. To the best of our knowledge, this is the first review to focus specifically on modulating gut microbiota through dietary therapies in LACPs. This paper systematically summarises the evidence on probiotic, prebiotic, and synbiotic interventions and their impact on gut microbial communities in individuals who are overweight and obese from LACPs. The aim of this review was to evaluate these therapies’ efficacy in modulating gut microbiota profiles in these individuals. Our findings may aid in improving intervention strategies and guide future research efforts to combat the growing prevalence of obesity among LACPs. While the authors acknowledge that probiotics exert effects beyond altering microbial profiles, this review specifically focused on this aspect to tackle obesity. Therefore, this was the primary focus of this investigation.

## Methods

This systematic review followed the 2020 Preferred Reporting Items for Systematic Reviews and Meta-Analyses (PRISMA) statement (Page et al., 2021) and the Cochrane Handbook for Systematic Reviews of Interventions (Higgins et al., 2023). The review protocol is registered with the International Prospective Register for Systematic Review (PROSPERO, ID: CRD42023493678) and available at www.crd.york.ac.uk/prospero/.

### Information Sources and Search Strategy

Literature searches of peer-reviewed publications were conducted in MEDLINE (via PubMed), Web of Science, Latin American and Caribbean Health Sciences Literature (LILACs), and the electronic database of clinical trials of the U.S. National Library of Medicine until May 2024. Search strings were designed following the Peer Review of Electronic Search Strategies (PRESS) guideline (McGowan et al., 2016) (Supplementary Table S1). The searches were restricted to English in all databases except for the LILACs database, which used Spanish. No publication date limits or automatic filters were applied. To achieve literature saturation, we employed a snowballing approach by reviewing the reference lists of the retrieved records to identify additional relevant studies.

### Eligibility criteria and study selection

Table 1 outlines the present review’s PICOS criteria (Population, Intervention, Comparison, Outcomes, and Study Design). To be eligible for inclusion, studies must have 1) been conducted on overweight or obese participants in LACPs, 2) investigated ingestion of probiotics, prebiotics or synbiotics, and 3) analysed gut microbiota composition compared to the control group or baseline. Studies that did not cover the impact of probiotics, prebiotics, or synbiotics on the alteration of gut microbiota in relation to obesity in LACPs or did not include microbiota composition analysis were exiled. Additionally, we excluded *in vitro* trials, preclinical and animal studies, and observational studies of multiple dietary interventions due to a lack of homogeneity and interference with pure effects of probiotic, prebiotic, and synbiotic consumption on gut microbiota composition. To ensure clinical similarity in the intervention’s nature and align with previous findings on the necessary intervention length for observable effects (Ishaque et al., 2018; Skrzydlo-Radomanska et al., 2021), we also excluded trials with fewer than 4 weeks of intervention. The studies defined overweight and obesity based on the body mass index (BMI). In accordance with the WHO classification criteria, individuals with a BMI of 25 kg/m^2^ or greater were considered eligible (World Health Organization, 2024). Three reviewers (L.L, E.F.V, and S.B) performed literature screening of titles and abstracts independently based on the previously described eligibility criteria, followed by full-text screening and discussion until agreement was reached between reviewers.

**Table 1.** PICOS criteria for inclusion of studies.

PICOS parameter	Inclusion criteria
Population	Overweight or obese participants in LACPs with no health conditions that potentially affect metabolism or body weight.
Intervention	Oral supplementation with probiotics, prebiotics or synbiotics isolated or included in a dietary plan.
Comparison	Comparisons of microbiota composition between the baseline and post-intervention or between different groups or arms (i.e. placebo vs. probiotics, prebiotics, and/or synbiotics).
Outcomes	Outcomes relating to faecal microbiota and gut bacteria composition.
Study design	Clinical trials, comparative studies, controlled clinical trials, randomised and non-randomised controlled trials.

### Data extraction and synthesis

The process for data extraction was carried out by two reviewers (E.F.V and L.L) and verified by one reviewer (M.M.B). An electronic data extraction form was developed using the 2024 Microsoft Excel software (Microsoft Corporation, 2023) to obtain the following information: author, population, study design, sample size, sample characteristics, intervention, methods of assessment, and outcome. As the main focus of this review, we extracted data on microbial profile changes, encompassing composition, abundance, and correlation with metabolites. Tables were constructed to summarise the characteristics of the studies, and a narrative synthesis of findings was performed following the Synthesis Without Meta-analysis (SWiM) guidelines (Campbell et al., 2020). Results regarding changes in microbial profile are presented with a phylogenetic tree created using diagram-generating software EdrawMax (version 13.0.2) (Edraw, 2024).

### Risk of Bias Assessment (RoB)

To assess the quality of the included studies, we used version 2 of The Cochrane risk-of-bias (RoB 2) tool for randomised control trials (RCTs) (Sterne et al., 2019). The domains of bias assessed using this tool are random sequence generation, allocation concealment, blinding of participants and personnel, incomplete outcome data, selective outcome reporting and other sources of bias. Finally, based on the assessment of each domain, the overall risk was judged as “low,” “some concerns,” or “high” following the Cochrane guidance.

## Results

**Figure 1.**
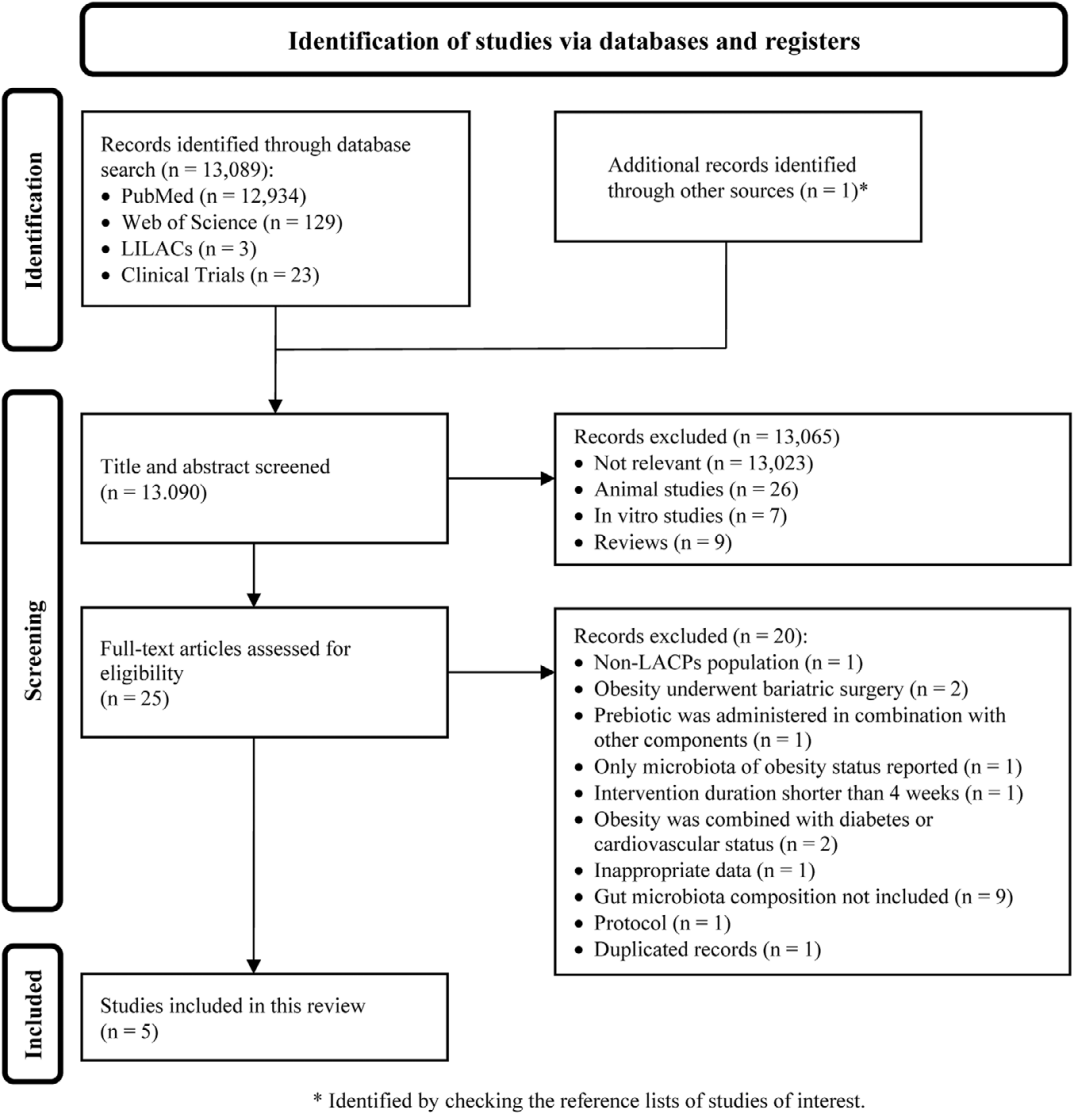
PRISMA flow diagram depicting the literature screening process. A total of 13,090 articles were identified in the initial search; 13,065 were removed after the title and abstract screening, and 25 remained for full-text review; of these, five were eligible for inclusion in the systematic review.

A comprehensive literature search of electronic databases and reference lists initially yielded 13,090 articles. After title and abstract screening, 13,065 articles were excluded because they were irrelevant to the review objective or not human studies. After a full-text evaluation of the 25 remaining records, five studies were eligible for inclusion in this review (Crovesy et al., 2021; Jamar et al., 2020; Martinez-Martinez et al., 2022; Peña et al., 2014; Ribeiro et al., 2023). The literature screening and selection processes and reasons for exclusion are shown in Figure 1.

### Characteristics of included trials

The five studies included in this analysis were conducted in Brazil (Crovesy et al., 2021; Jamar et al., 2020; Ribeiro et al., 2023), Mexico (Martinez-Martinez et al., 2022), and Chile (Peña et al., 2014). Each study provided dietary treatment with probiotics, prebiotics, or synbiotics to participants who were overweight or obese. The sample sizes of the trials ranged from 21 to 39 participants, with a total of 164 participants aged between 6 and 59 years. Three studies included men and women (Jamar et al., 2020; Peña et al., 2014; Ribeiro et al., 2023), one trial recruited children of both sexes (Martinez-Martinez et al., 2022), and one focused on adult women (Crovesy et al., 2021). All the reviewed trials used a parallel RCT design. No adverse effects of oral supplementation were reported in any of the RCTs. Interventions in the included RCTs evaluated two forms of probiotics: *Bifidobacterium lactis* UBBLa-70 (Crovesy et al., 2021) and *Lactobacillus casei* Shirota (Martinez-Martinez et al., 2022). Prebiotic supplementation was administered in two studies utilising locally sourced functional foods with potential prebiotic properties. Namely, studies used yacon flour (*Smallanthus sonchifolius*) (Ribeiro et al., 2023) and juçara berry fruit (*Euterpe edulis* Martius), which is a native species of the Atlantic Forest/Brazil and a promising source of antioxidants, mainly anthocyanins (Jamar et al., 2020). Additionally, synbiotic supplementation was explored in three studies using combinations such as *B. lactis* UBBLa-70 and FOS (Crovesy et al., 2021), *L. casei* Shirota with either inulin or fructans from *Agave salmiana* (Martinez-Martinez et al., 2022), and *B. lactis* Bb12 mixed with oligofructose (Peña et al., 2014). Overall, the intervention duration across the included trials ranged from 6 to 8 weeks. Table 2 displays the characteristics of the included studies and details of the applied treatments.

**Table 2.** Characteristics of the studies and summary of probiotic, prebiotic, and synbiotic interventions with the reported gut microbiome changes among the included trials (*n* = 5).

Reference	RCT design(blinding)	Sample size	Participants’ characteristics	Intervention				Microbial community identification method
				Biotic	Treatment (no.)	Control (no.)	Dosage /duration	
**Brazil**								
Crovesy et al. (2021)	Parallel (Yes)	*n* = 32	Women, obese BMI: 30–34.9 kg/m^2^ Age:Probiotics 34.70 ± 8.99, Synbiotics 35.18 ± 5.58	Probiotics	10^9^ CFU *Bifidobacterium lactis* UBBLa–70 + 5 g MD + low-energy diet, (*n* = 10)	Placebo: gelatine +5 g MD, (*n* = 11)	1/day 8 weeks	Real time-PCR
				Synbiotics	10^9^ CFU *B. lactis* UBBLa–70 + 5 g FOS + low-energy diet, (*n* = 11)			
Jamar et al. (2020)	Parallel (Yes)	*n* = 35	Adults, obese BMI: ≥ 30 ≤ 39.9 kg/m^2^ Age: 31–59 years	Prebiotics	5 g of lyophilized juçara pulp, (*n* = 18)	Placebo: 5 g MD, (*n* = 17)	1/day 6 weeks	Quantitative PCR
Ribeiro et al. (2023)	Parallel (Yes)	*n* = 21	Adults, obese BMI: 25–34.9 kg/m^2^ Age: 20–95 years	Prebiotic	Breakfast drink containing 25 g yacon flour (8.7 g FOS) + energy-restricted diet (*n* = 11)	Control Breakfast drink: 0 g yacon flour (0 g FOS), (*n* = 10)	1/day 6 weeks	16S rRNA gene sequencing
**Mexico**								
Martinez-Martinez et al. (2022)	Parallel (Yes)	*n* = 38	Children, overweight, obese, and healthy Age: 6–10 years	Synbiotics	15 mL fermented milk containing *Lactobacillus casei* strain Shirota (10^8^ CFU/mL) + 3 g of inulin (*n* = 13)	Probiotic: 15 mL fermented milk containing *L. casei* strain Shirota (10^8^ CFU/mL) (*n* = 12)	2/day, school days only 6 weeks	16S rRNA gene sequencing
				Synbiotics	15 mL fermented milk containing *L. casei* strain Shirota (10^8^ CFU/mL) + 3 g of *Agave salmiana*, (*n* = 13)			
**Chile**								
Pena et al. (2014)	Parallel (Yes)	*n* = 38	Adults, obese BMI: 36.7 ± 5.3 kg/m^2^ Age: 34.8 ± 9.2 years	Synbiotics	1 g lyophilized *B. lactis* Bb12 (10^10^ CFU/g) + 8 g oligofructose, (*n* = 18)	Placebo: 9 g MD (*n* = 20)	2/day 6 weeks	Quantitative PCR

**Abbreviations:** AGEs, advanced glycation end products; BMI, body mass index; CFU, colony-forming unit; EGPs, early glycation products, FOS, fructooligosaccharide; MD, maltodextrin; PCR, polymerase chain reaction; RCT, randomised controlled trial; SCFAs, short-chain fatty acid.

### Risk of bias assessment and methodological quality

**Figure 2.**
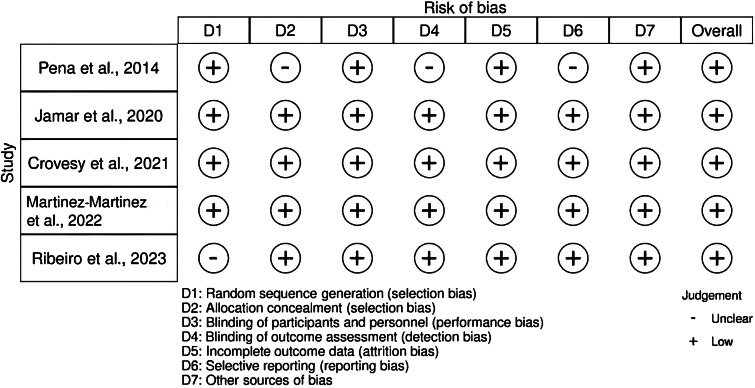
Risk of bias assessment of the included studies using the RoB 2 tool.

Five RCTs were assessed for quality using the RoB 2 tool. All trials showed either a low or unclear RoB across the seven domains of this tool, leading to an overall judgement of low RoB for all included studies (Figure 2, Supplementary Table S2). We further evaluated the quality of technologies employed for collecting and handling faecal samples across studies to gain insights into the analysis and reporting methods for gut microbiota composition (Supplementary Table S3). Overall, differences in the analysis and reporting methods were observed among the five RCTs, particularly in the methods used to investigate the DNA levels of intestinal bacteria. Two studies employed real-time polymerase chain reaction (PCR) with corresponding primers and SYBR Green PCR Master Mix (Applied Biosystems) to identify the relative DNA levels of intestinal bacteria via amplification (Jamar et al., 2020; Peña et al., 2014). One study evaluated the phyla and class levels of bacteria performing real-time PCR with the StepOne Plus Real-Time PCR System (Life Technologies) (Crovesy et al., 2021). Another study reported the relative abundance of bacterial taxa regarding operational taxonomic units and amplicon sequence variants (Martinez-Martinez et al., 2022).

### Results of individual trials

Despite the increasing interest in the potential effects of probiotics, prebiotics, and synbiotics on gut microbiota composition in individuals who are overweight and obese, the available literature is limited. Only five studies met our inclusion criteria, indicating a notable gap in research in this field. Among the five included studies, only one RCT explored the effects of probiotic intervention on microbial communities (Crovesy et al., 2021). This particular study focused on a group of 10 Brazilian women with obesity. Over 8 weeks, participants were supplemented with probiotics at a dosage of 10^9^ CFU *B. lactis* UBBLa-70/day. Correlation analysis conducted on changes in metabolites and phyla following the probiotic supplementation showed negative associations of the phylum Verrucomicrobia with lactate and lipids, and a positive association with isoleucine when compared with the baseline. Moreover, negative correlations were found between changes in Firmicutes phylum and lipids (VLDL, LDL, and (CH_2_)*_n_* lipids) (Crovesy et al., 2021). In the prebiotic context, two Brazilian studies have reported post-intervention changes in the microbial population of adults with obesity. Supplementation with 5 g of lyophilised juçara pulp daily for 6 weeks led to a significant increase in the relative abundance of *Akkermansia muciniphila* (*p* = 0.003), *Bifidobacterium* spp. (*p* < 0.001), and *Clostridium coccoides* (*p* < 0.001) species, but not for *Lactobacillus* spp., regardless of dietary fibre intake (Jamar et al., 2020). In a similar intervention duration, the daily consumption of a breakfast beverage containing 25 g of yacon flour (8.7 g FOS) with an energy-restricted diet resulted in notable changes at the genera level. An increase in *Bifidobacterium*, *Blautia*, *Subdoligranulum*, and *Streptococcus* was observed compared with the control group, but no changes were noted in the Firmicutes/Bacteroidetes ratio (Ribeiro et al., 2023). Moreover, negative associations were found between the concentrations of advanced glycation end-products and early glycation products and relative abundances of the genera *Ruminococcus* and *Prevotella*, respectively. In addition, a positive correlation was observed between the concentrations of SCFAs and *Coprococcus* and *Howardella.*

**Figure 3.**
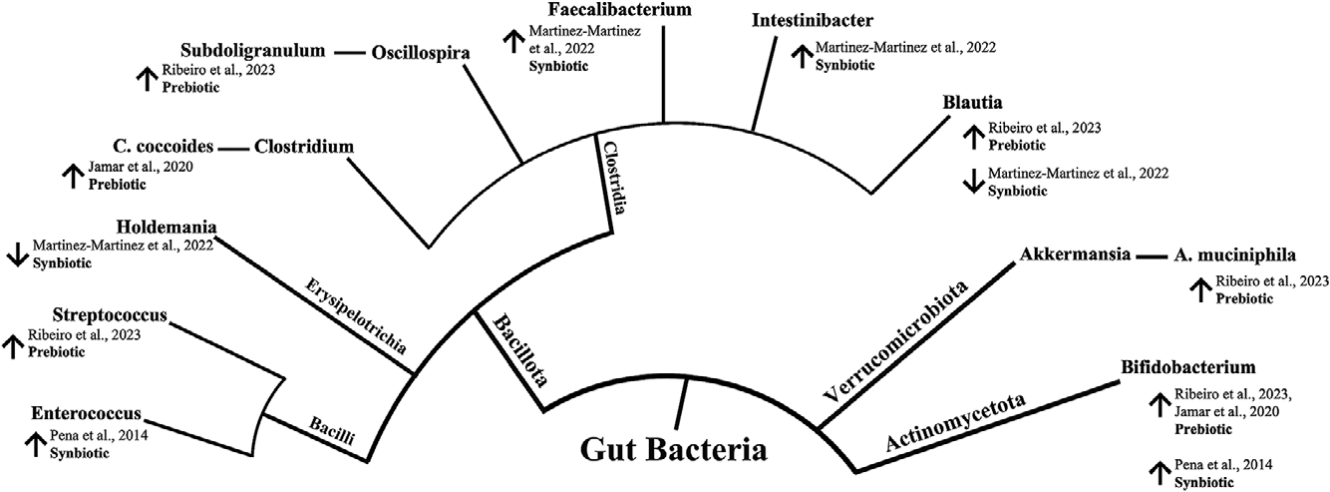
Reported changes in gut microbiota following prebiotic and synbiotic supplementation among overweight and obese participants from LACPs. The inner circle represents the phylum level, whereas the outer circle represents the genus level. An upward arrow (↑) indicates increased abundance compared to baseline or control, while a downward arrow (↓) indicates decreased abundance compared to baseline or control.

A Brazilian study investigated the effects of a synbiotic intervention on a group of 11 women with obesity. The participants were provided sachets containing 10^9^ CFU *B. lactis* UBBLa-70 and 5 g of FOS to be administered daily for 8 weeks. The study found a positive correlation between changes in the *Bacteroidetes* genus and serum glycerol (Crovesy et al., 2021). Compared with baseline measurements, this intervention resulted in unique metabolite changes, including increased levels of pyruvate and alanine and decreased levels of citrate and branched-chain amino acids. Additionally, within an intervention group comprising 13 Mexican children who were overweight and obese (categorised as ≥85th percentile and ≥ 95th percentile for BMI-for-age, respectively), supplementation with synbiotics was administered twice daily for 6 weeks (Martinez-Martinez et al., 2022). The synbiotic dose contained 3 g of fructans from *A. salmiana*, added to a primary base of 15 mL fermented milk and *L. casei* (10^8^ CFU/mL). The results showed significant stimulation in microbiota abundance and diversity. In particular, a significant impact on *Faecalibacterium* and *Holdemanella* (*p* = 0.00151) was detected compared with the group pre-treatment. Moreover, an RCT including 38 adults with obesity from Chile examined the effects of synbiotic treatment: 18 participants received the treatment and 20 received a placebo. The synbiotic treatment comprised administering 8 g of oligofructose with 1 g of lyophilised *B. lactis* BB12 (10^10^ CFU/g) twice daily for 6 weeks. This treatment significantly increased the concentration of *Enterococcus* (*p* = 0.006) and the proportion of *Bifidobacterium* (*p* = 0.049), without affecting other studied bacterial populations, such as *Lactobacillus* and *Bacteroides* (Peña et al., 2014). Although alterations in body weight were not the primary focus of the included studies, changes following biotic interventions were generally inconspicuous (Peña et al., 2014) or exhibited no statistically significant differences between the intervention groups (Crovesy et al., 2021; Martinez-Martinez et al., 2022). However, one study conducted in Brazil reported that participants in the yacon flour intervention group (*n* = 11) experienced a notable reduction in weight compared with the control group following a 6-week intervention period (Ribeiro et al., 2023). Figure 3 shows a summary of the reported changes in gut microbiota following prebiotic and synbiotic supplementation among participants who are overweight and obese across the included studies.

## Discussion

This review provides a comprehensive overview of current evidence on the effects of probiotics, prebiotics, and synbiotics on the gut microbiota profiles of overweight and obese individuals from LACPs, specifically highlighting records from Brazil, Chile, and Mexico. Previous systematic reviews have focused primarily on the effects of these oral supplements on biochemical parameters and anthropometric changes, rather than on microbial stimulation (Alvarez-Arrano and Martin-Pelaez, 2021; Borgeraas et al., 2018; Hadi et al., 2019a; Shirvani-Rad et al., 2021; Suzumura et al., 2019; Zhang et al., 2016). Thus, we aimed to elucidate the effects of probiotic, prebiotic, and synbiotic usage on gut microbiota composition in LACPs to evaluate the effectiveness of these nutritional therapeutic approaches for modulating gut microbiota profiles. The human gut microbiota is characterised by core communities of bacteria that remain stable over time and are associated with long-term diet (Bayer et al., 2020). Dietary strategies incorporating probiotics and prebiotics can affect microbiome composition (Marchesi et al., 2016). Obesity, a major health issue, predisposes individuals to cardiometabolic disorders, such as cardiovascular diseases and type 2 diabetes mellitus (Zhang et al., 2016). Multiple systematic reviews with meta-analyses have demonstrated that short-term consumption of probiotics, prebiotics, and synbiotics has a favourable effect on obesity indicators, such as weight and BMI (Alvarez-Arrano and Martin-Pelaez, 2021; Borgeraas et al., 2018; Rasaei et al., 2024; Zhang et al., 2016). However, conflicting results have also emerged from the literature (Suzumura et al., 2019). Remarkably, the anti-obesity effects of probiotics may be more pronounced in individuals who are overweight or obese compared with those of normal weight (Zhang et al., 2016). Various mechanisms impact the potential of biotic supplementation’s effect on body weight. The production of SCFAs during prebiotic fermentation beneficially influences energy metabolism, appetite regulation, and insulin sensitivity, contributing to these effects (Rasaei et al., 2024). Additionally, the proliferation of beneficial bacteria facilitated by probiotics and prebiotics supports the restoration of epithelial cell tight junctions, leading to decreased intestinal permeability, reduced microbial translocation, and lower endotoxin-induced inflammation (Alvarez-Arrano and Martin-Pelaez, 2021). This reduction in inflammation, combined with the modulation of gut hormones through biotic treatment, enhances hypothalamic insulin sensitivity, improves satiety, and ultimately decreases food intake (Carvalho and Saad, 2013). Moreover, probiotic supplementation holds promise for modulating inflammatory cytokines synthesis through interactions with the immune system (Cristofori et al., 2021). Nevertheless, except for one study (Ribeiro et al., 2023), the effect on body weight was either negligible or not reported in the included study, as identified in our systematic review.

Another proposed mechanism by which biotic supplementation potentially affects obesity is gut microbiome modulation (Hadi et al., 2020b). Probiotics, such as *Bifidobacterium* and *Lactobacillus* strains, have been reported to have multiple interactions with the host (Marchesi et al., 2016). Analysis of the current review showed that probiotic interventions involving *B. lactis* resulted in significant changes in metabolite profiles, which correlated with changes in Firmicutes and Verrucomicrobia phyla (Crovesy et al., 2021). However, researchers have suggested that these effects could be due to the influence of the accompanying low-energy diet, regardless of changes in the gut microbiota. Furthermore, probiotic supplementation was examined as a control treatment and compared with synbiotic treatment. Post-intervention gut bacterial changes were reported in the synbiotic groups; however, data from the probiotic groups were not included (Martinez-Martinez et al., 2022). Clinical studies have yielded inconsistent results regarding changes in specific microbial communities following probiotic interventions (Kristensen et al., 2016; Lahtinen et al., 2012; Larsen et al., 2011, 2013; Plaza-Diaz et al., 2015). Highlighting that intestinal microbial population alteration is not the only anti-obesity effect of probiotics is important. Even without the observed changes in faecal microbiota, probiotics can still exert modification effects through the previously mentioned alternative mechanisms, such as altering SCFA production or microbial activities (Olvera-Rosales et al., 2021; Sanchez et al., 2017).

The present findings revealed beneficial alterations in gut microbiota composition through supplementation with prebiotics and synbiotics among LACP adults who are overweight and obese, with a substantial increase in health-promoting genera (Figure 3). The synbiotic intervention, involving *L. casei* combined with fructans from *A. salmiana*, in Mexican children who are obese and overweight stimulated microbiota abundance and diversity, significantly increasing *Faecalibacterium* and *Intestinibacter* and decreasing the *Holdemanella* and *Blautia* genera (Martinez-Martinez et al., 2022). In particular, *Faecalibacterium* has been suggested to indicate intestinal health due to its anti-inflammatory effects and its reduced prevalence in numerous gastrointestinal and metabolic disorders (Martin et al., 2023; Miquel et al., 2013). Moreover, the *Holdemanella* genus is recognised as a potential health contributor. It has been positively correlated with the intake of fermented dairy products, carbohydrates, and dietary fibre (Ma et al., 2021; Zhang et al., 2022). In a related study, the influence of probiotics on gut microbiota modulation was investigated. Lactic acid bacteria (*Lactobacillus acidophilus*, *Lactiplantibacillus plantarum*, *Limosilactobacillus fermentum*, and *Lactobacillus delbrueckii*) were administered to participants with obesity for 2 weeks. This study reported a decrease in the abundance of the *Holdemanella* genus, while concurrently noting an increase in the *Blautia* genus (Burakova et al., 2022). Our data showed an increase in *Bifidobacterium* following the synbiotic administration of *B. lactis* BB 12 mixed with oligofructose in patients with obesity patients from Chile (Peña et al., 2014). In a comprehensive review of both animal and human studies, researchers investigated the impact of probiotics and prebiotics on the relationship between intestinal microbiota and obesity, revealing a predominant modulatory effect characterised by increased *bifidobacteria*, frequently accompanied by weight reduction and improvements in obesity-related indicators (da Silva et al., 2013). Additionally, a lower number of *Bifidobacterium* has been noted in individuals who are obese and overweight, implying that this genus is inversely linked to obesity and its comorbidities (Delzenne et al., 2011; Schwiertz et al., 2010). Nonetheless, the effect of *Bifidobacterium* on body weight is suggested to be strain-specific. Different strains may exert their effects through several mechanisms, including the regulation of bile acid metabolism, SCFA production, and protection from metabolic endotoxaemia (Brusaferro et al., 2018).

In the context of prebiotics, microbiota balance or activities are modified by prebiotics, oligofructose, galacto-oligosaccharides, FOS, inulin, and lactulose. They have been frequently reported to increase populations of *Bifidobacterium* and *Lactobacillus* (Macfarlane et al., 2006). Two studies in this systematic review explored the effects of prebiotics on the microbial profiles of participants who are overweight and obese (Jamar et al., 2020; Ribeiro et al., 2023). A lyophilised juçara fruit pulp, which previously showed a bifidogenic effect in reshaping the microbiota in an animal model (Jamar et al., 2018), was used for its potential prebiotic function. In Brazilian adults with obesity, this treatment increased the abundance of *A. muciniphila*, *Bifidobacterium* spp., and *C. coccoides* (Jamar et al., 2020). These species are associated with positive effects on their hosts (Gómez-Gallego et al., 2016). In particular, a higher abundance of *A. muciniphila* has been linked to healthier metabolic status and enhanced glucose homeostasis, blood lipids, and body composition following calorie restriction in adults who are overweight and obese (Brusaferro et al., 2018; Dao et al., 2016; Everard et al., 2013). Moreover, *C. coccoides* may benefit host health by the abundant production of SCFAs (Scott et al., 2014). Prebiotic intervention with yacon flour comprising FOS also resulted in a potentially positive shift in gut microbiota composition in Brazilian participants with obesity by increasing the abundance of *Bifidobacterium* and the Firmicutes-belonging genera: *Blautia*, *Subdoligranulum*, and *Streptococcus* (Ribeiro et al., 2023). The *Blautia* and *Subdoligranulum* groups contain various butyrate-producing bacteria (Holmstrom et al., 2004; Wan et al., 2019). Increased *Blautia* abundance has been suggested to have a favourable effect on blood lipid profiles and indicators associated with excess body weight (Ribeiro et al., 2023; Upadhyaya et al., 2016). However, we noted contrasting findings regarding the impact of synbiotic and prebiotic interventions on this genus (Martinez-Martinez et al., 2022; Ribeiro et al., 2023), possibly attributed to differences in participants’ characteristics (adults vs. children), complementary diet (regular vs. energy-restricted), substrate, or bacteria composition.

Recognising the limitations of the identified studies and addressing critical gaps in the existing literature is essential. The small sample sizes and lack of high-throughput technologies for microbiota evaluation require replication of these studies with larger sample sizes. Moreover, employing metagenomics or sequencing technologies, which enable strain-level resolution, will provide more detailed insights into the microbiota. Furthermore, the evaluation of the quality of technologies used across included studies revealed significant heterogeneity in the analyses and reporting of microbial outcomes. These methodological differences make direct comparisons of findings challenging. Variations in the primers used to target the 16S rRNA region of microbial DNA and the use of different databases to assign taxonomies further limited the ability to compare results between studies. Nevertheless, information regarding the baseline-predominant microbiota observed in populations of LACP individuals who are overweight or obese was not addressed in the included studies.

Another limitation of the included studies was the diverse forms in which probiotics, prebiotics, and synbiotics were administered. These forms included capsules (Crovesy et al., 2021), powder dissolved in a breakfast drink (Ribeiro et al., 2023), lyophilised powder ingested alone (Jamar et al., 2020; Peña et al., 2014), and mixtures with fermented milk (Martinez-Martinez et al., 2022). Various food formulations, mechanical processing methods, and modes of administration play a crucial role in determining the quantity and composition of substances that reach the gut bacteria. This, in turn, affects bacterial growth and microbiota metabolite production (Ercolini and Fogliano, 2018; Kristensen et al., 2016). For instance, different probiotic formulations have been suggested to vary in their efficiency and capacity to provide viable functional bacteria in sufficient numbers to produce health benefits (Govender et al., 2014). Furthermore, drawing firm conclusions from the current review is challenging due to the diversity in age and sex among the study participants, which range from young children to adults. Despite analysing a limited number of studies, we noted substantial heterogeneity in bacterial genera, supplement forms, doses, study populations, and dietary contexts. Our observation aligns with the findings of a previous systematic review (Hadi et al., 2021), indicating that these challenges are persistent and complex to address. This may have contributed to the variability in the outcomes, considering the differences in the participants’ microbiota. Nevertheless, less variation was observed in intervention duration. In our review, a short-term 6-week intervention successfully demonstrated the impact of prebiotic and synbiotic treatments on microbial profiles. Researchers have tended to explore the bifidogenic properties and effectiveness of native dietary plants such as *A. salmiana*, juçara berry, and yacon (Crovesy et al., 2021; Jamar et al., 2020; Martinez-Martinez et al., 2022). Such findings facilitate the development of culturally specific, low-cost nutritional therapies for gut microbiota modulation in LACP communities’ individuals with obesity. However, follow-up studies are required to determine the effects of these changes on obesity.

This is the first systematic review to deliver a comprehensive overview of available evidence on the effects of probiotic, prebiotic, and synbiotic supplementation on gut microbiota profiles, particularly focusing on LACPs. This review’s strengths include using PRISMA guidelines (Page et al., 2021) and the Cochrane Handbook for Systematic Reviews of Interventions (Higgins et al., 2023) to design a solid search strategy and minimise biases. The quality of evidence was evaluated using the Cochrane risk-of-bias tool for RCTs (Sterne et al., 2019). We also reviewed the quality and methodologies used in microbiota analysis in these studies. Our results have significant implications for clinical practice and future research, offering valuable insights for healthcare policies and guiding intervention development. While our systematic review aimed to comprehensively synthesise the existing literature, acknowledging certain limitations, including the small number of included studies, is essential. Only five RCTs were identified from three countries: Brazil, Mexico, and Chile. The scarcity of eligible studies may limit the generalisability of the findings and depth of the analysis. Second, the average sample size of the included studies was 33 participants, with the number of participants per intervention group ranging from 10 to 18. This sample size potentially decreases the accuracy of the reported effects. In addition, our ability to draw sex-specific conclusions regarding the impact of biotic treatments on gut microbial profiles was limited, as most studies included both men and women. Only one study exclusively focused on women. Our investigation found that in most studies, changes in microbial profiles were examined as secondary outcomes, with only one study treating them as primary outcomes. This discrepancy highlights the importance of acknowledging the secondary status of this variable in the included studies, which may be a limitation of the present review. Finally, our research primarily focused on alterations in bacterial composition. However, these changes should not be regarded as the only indicators of the health potential of biotic interventions in individuals with excessive body weight. An examination of additional gut microbiota modulation markers and obesity-related indicators would have enhanced the comprehensiveness of our analysis, leading to a more conclusive determination of the effects of biotic interventions in participants with obesity. Hence, future studies should include alterations in the gut microbiota as the primary outcome, while considering other relevant factors. This approach will enhance our understanding of the mechanisms by which probiotics, prebiotics, and synbiotics modulate gut microbiota profiles and improve the host’s metabolic health. The effects of confounding variables including age, lifestyle, and disease conditions remain ambiguous. In some included studies, participants adhered to a low-energy diet during the intervention (Crovesy et al., 2021; Ribeiro et al., 2023), whereas in others, they maintained a normal diet (Jamar et al., 2020; Martinez-Martinez et al., 2022; Peña et al., 2014). This variability underscores the importance of examining the impact of functional foods on gut microbiota composition independent of concurrent dietary changes, as these directly influence the results. Future crossover design RCTs with larger sample sizes are warranted in different LACP countries. These studies should be focused on detecting significant changes in gut microbiota composition and establishing firm conclusions regarding the efficacy of probiotics, prebiotics, and synbiotics in modulating host microbial profiles. Moreover, elucidating the underlying mechanisms of action of these dietary interventions is essential.

## Conclusion

Our analysis revealed a lack of adequate data to draw definitive conclusions on the impact of probiotics, prebiotics, and synbiotics on the gut microbiota profiles of LACP individuals who are overweight/obese. These limitations stem from methodological heterogeneity and the absence of high-throughput technologies for microbiota evaluation, which hinder the observation of biotic effects on gut microbiota. The observed discrepancies in reported outcomes may be attributed to variations in supplement formulation, dosage, and population characteristics. To address these gaps, further parallel RCTs with larger sample sizes are needed, focusing on dietary therapeutic interventions aimed at modulating gut microbiota imbalances and elucidating their implications in managing metabolic conditions. Additionally, adopting metagenomic or sequencing technologies for strain-level resolution is crucial for enhancing the precision of gut microbiota analysis and reporting methods in future research. Targeting gut microbiota represents a potentially cost-effective nutritional strategy for managing obesity and its associated disorders in LACPs.

## Supporting information

Bineid et al. supplementary material 1 Bineid et al. supplementary material

Bineid et al. supplementary material 2 Bineid et al. supplementary material

## Data Availability

The original contributions presented in this study are included in the article/Supplementary Material, and further enquiries can be directed to the corresponding author.

## Supplementary material

The supplementary material for this article can be found at http://doi.org/10.1017/gmb.2024.12.
