# Supplementary material for: The effect of probiotics, prebiotics and synbiotics on gut microbial community profile in overweight and obese Latin American and Caribbean populations: a systematic review of human trials
Source: Gut Microbiome (Camb). 2025 Jan 17;6:e2. doi: 10.1017/gmb.2024.12 (PMC11810601; doi:10.1017/gmb.2024.12)
Supplement: Bineid et al. supplementary material 1 — Bineid et al. supplementary material [file S2632289724000124sup001.docx]

**Supplementary Materials**

**Table S 1.** Search Strings in Different Databases

| **PubMed** |
| --- |
| ((Latin America* OR Caribbean OR Belize OR Costa Rica* OR Cuba* OR Dominican Republic OR El Salvador OR Guatemala* OR Haiti* OR Hondura* OR Jamaica* OR Mexic* OR Nicaragua* OR Panama* OR Saint Lucia* OR South America* OR Argentin* OR Bolivia* OR Brazil* OR Chile* OR Colombia* OR Ecuador* OR Paraguay* OR Peru* OR Uruguay* OR Venezuela*) AND (gut microbiota OR microbiome OR firmicutes OR Bacteroidetes OR actinobacteria OR proteobacteria OR prevotella OR ruminococcus OR saccharomyces OR probiotic* OR pro-biotics OR probiotic-supplement OR lactobacillus OR Bifidobacterium OR yeast* OR live bacteria OR yogurt OR kefir OR sauerkraut OR kimchi OR kombucha OR symbiotic) AND (Obesity OR obese OR overweight BMI OR “body mass index” OR WHR OR “waist to hip ratio” OR “waist-hip ratio” OR skinfold OR adiposity OR fat percentage OR “body fat mass” OR WC OR “waist circumference” OR “weight loss” OR “weight gain” OR weight OR HC OR “hip circumference”) AND (trial) NOT (animal OR pig OR mouse OR rat OR in vitro OR in silico OR “observational OR case-control))  ((Latin America* [tiab] OR Caribbean [tiab] OR Belize [tiab] OR Costa Rica* [tiab] OR Cuba* [tiab] OR Dominican Republic [tiab] OR El Salvador [tiab] OR Guatemala* [tiab] OR Haiti* [tiab] OR Hondura* [tiab] OR Jamaica* [tiab] OR Mexic* [tiab] OR Nicaragua* [tiab] OR Panama* [tiab] OR Saint Lucia* [tiab] OR South America* [tiab] OR Argentin* [tiab] OR Bolivia* [tiab] OR Brazil* [tiab] OR Chile* [tiab] OR Colombia* [tiab] OR Ecuador* [tiab] OR Paraguay* [tiab] OR Peru* [tiab] OR Uruguay* [tiab] OR Venezuela* [tiab]) AND (“gut microbiota” OR microbiome OR firmicutes OR Bacteroidetes OR actinobacteria OR proteobacteria OR prevotella OR ruminococcus OR saccharomyces OR probiotic* OR pro-biotics OR probiotic-supplement OR lactobacillus OR lactobacilli OR boulardii OR coagulans OR GOS OR FOS OR XOS OR Inulin OR fructans OR enterocci OR enterococcus OR Bifidobacterium OR yeast* OR “live bacteria” OR “probiotic food*” OR yogurt OR kefir OR sauerkraut OR kimchi OR kombucha OR symbiotic* OR “symbiotic food*” OR synbiotic OR oligosaccharides OR fructooligosaccharide OR xylooseoligosaccharide OR Galacto-oligosaccharides OR galactooligosaccharides OR chicory OR yacon roots OR artichoke ) AND (Obesity [tiab] OR obese [tiab] OR overweight [tiab] BMI [tiab] OR "body mass index" [tiab] OR WHR [tiab] OR "waist to hip ratio" [tiab] OR "waist-hip ratio" [tiab] OR skinfold [tiab] OR adiposity [tiab] OR fat percentage [tiab] OR "body fat mass" [tiab] OR WC [tiab] OR "waist circumference" [tiab] OR "weight loss" [tiab] OR "weight gain" [tiab] OR weight [tiab] OR HC [tiab] OR "hip circumference"[tiab]) AND (trial OR intervention OR “clinical study” OR double blind RCT OR randomized control trial OR NRCT OR single-arm OR crossover OR parallel) NOT (animal OR pig OR mouse OR rat OR in vitro OR silico OR cross-sectional [tiab])) |
| **Web of Science** |
| Latin America* OR Caribbean OR Belize OR Costa Rica* OR Cuba* OR Dominican Republic OR El Salvador OR Guatemala* OR Haiti* OR Hondura* OR Jamaica* OR Mexic* OR Nicaragua* OR Panama* OR Saint Lucia* OR South America* OR Argentin* OR Bolivia* OR Brazil* OR Chile* OR Colombia* OR Ecuador* OR Paraguay* OR Peru* OR Uruguay* OR Venezuela* (Abstract) and “gut microbiota” OR microbiome OR firmicutes OR Bacteroidetes OR actinobacteria OR proteobacteria OR prevotella OR ruminococcus OR saccharomyces OR probiotic* OR pro-biotics OR probiotic-supplement OR lactobacillus OR lactobacilli OR boulardii OR coagulans OR GOS OR FOS OR XOS OR Inulin OR fructans OR enterococci OR enterococcus OR Bifidobacterium OR yeast* OR “live bacteria” OR “probiotic food*” OR yogurt OR kefir OR sauerkraut OR kimchi OR kombucha OR symbiotic* OR “symbiotic food*” OR synbiotic OR oligosaccharides OR fructooligosaccharide OR xylooseoligosaccharide OR Galacto-oligosaccharides OR galactooligosaccharides OR chicory OR yacon roots OR artichoke (All Fields) and Obesity OR obese OR overweight BMI OR “body mass index” OR WHR OR “waist to hip ratio” OR “waist-hip ratio” OR skinfold OR adiposity OR fat percentage OR “body fat mass” OR WC OR “waist circumference” OR “weight loss” OR “weight gain” OR weight OR HC OR “hip circumference” (All Fields) and trial OR intervention OR “clinical study” OR double blind RCT OR randomized control trial OR nact OR single-arm OR crossover OR parallel (All Fields) not animal OR pig OR mo. use OR rat OR in vitro OR silico OR cross-sectional (Abstract) |
| **LILACs** |
| América Latina* OR Caribe OR Belice OR Costa Rica* OR Cuba* OR República Dominicana OR El Salvador OR Guatemala* OR Haití* OR Honduras* OR Jamaica* OR México* OR Nicaragua* OR Panamá* OR Santa Lucía* OR América del Sur* OR Argentina* OR Bolivia* OR Brasil* OR Chile* OR Colombia* OR Ecuador* OR Paraguay* OR Perú* OR Uruguay* OR Venezuela*  Obesidad OR obeso OR sobrepeso OR IMC OR índice de masa corporal OR RCC OR "indice cintura-cadera" OR "indice cintura-cadera" OR pliegue cutáneo OR adiposidad OR porcentaje de grasa OR "masa de grasa corporal " OR WC OR "circunferencia de la cintura" OR "pérdida de peso" OR "aumento de peso" OR peso OR HC OR "circunferencia de la cadera"  **(**probiótico) AND (brasil) AND (Obesidad OR obeso OR sobrepeso OR IMC OR índice de masa corporal OR RCC OR "indice cintura-cadera" OR "indice cintura-cadera" OR pliegue cutáneo OR adiposidad OR porcentaje de grasa OR "masa de grasa corporal " OR WC OR "circunferencia de la cintura" OR "pérdida de peso" OR "aumento de peso" OR peso OR HC OR "circunferencia de la cadera")  (probiótico) AND (mexic*) AND (Obesidad OR obeso OR sobrepeso OR IMC OR índice de masa corporal OR RCC OR "indice cintura-cadera" OR "indice cintura-cadera" OR pliegue cutáneo OR adiposidad OR porcentaje de grasa OR "masa de grasa corporal " OR WC OR "circunferencia de la cintura" OR "pérdida de peso" OR "aumento de peso" OR peso OR HC OR "circunferencia de la cadera")  (probiótico) AND (argentin*) AND (Obesidad OR obeso OR sobrepeso OR IMC OR índice de masa corporal OR RCC OR "indice cintura-cadera" OR "indice cintura-cadera" OR pliegue cutáneo OR adiposidad OR porcentaje de grasa OR "masa de grasa corporal " OR WC OR "circunferencia de la cintura" OR "pérdida de peso" OR "aumento de peso" OR peso OR HC OR "circunferencia de la cadera")  For the rest of countries  (prebiótico) AND (country) AND (Obesidad OR obeso OR sobrepeso OR IMC OR índice de masa corporal OR RCC OR "indice cintura-cadera" OR "indice cintura-cadera" OR pliegue cutáneo OR adiposidad OR porcentaje de grasa OR "masa de grasa corporal " OR WC OR "circunferencia de la cintura" OR "pérdida de peso" OR "aumento de peso" OR peso OR HC OR "circunferencia de la cadera")  (simbiotico) AND (country) AND (Obesidad OR obeso OR sobrepeso OR IMC OR índice de masa corporal OR RCC OR "indice cintura-cadera" OR "indice cintura-cadera" OR pliegue cutáneo OR adiposidad OR porcentaje de grasa OR "masa de grasa |
| **The electronic database of clinical trials of the U.S. National Library of Medicine** |
| Country: Belize OR Costa Rica* OR Cuba* OR Dominican Republic OR El Salvador OR Guatemala* OR Haiti* OR Hondura* OR Jamaica* OR Mexic* OR Nicaragua* OR Panama* OR Saint Lucia* OR South America* OR Argentin* OR Bolivia* OR Brazil* OR Chile* OR Colombia* OR Ecuador* OR Paraguay* OR Peru* OR Uruguay* OR Venezuela  probiotics \| Obesity \| Mexico  prebiotics \| Obesity \| Mexico  synbiotics \| Obesity \| Mexico  probiotics \| Obesity \| Brazil  prebiotics \| Obesity \| Brazil  synbiotics \| Obesity \| Brazil  probiotics \| Obesity \| Chile  prebiotics \| Obesity \| Chile  synbiotics \| Obesity \| Chile |

| **First author (publication year)** | **Domains** | | | | | | | **Overall quality** |
| --- | --- | --- | --- | --- | --- | --- | --- | --- |
|  | **Random sequence generation** | **Allocation concealment** | **Blinding of participants and personnel** | **Blinding of outcome assessment** | **Incomplete outcome data** | **Selective reporting** | **Other sources of bias** |  |
| Pena et al. (2014) | Low | Unclear | Low | Unclear | Low | Unclear | Low | Good |
| Jamar et al. (2020) | Low | Low | Low | Low | Low | Low | Low | Good |
| Crovesy et al. (2021) | Low | Low | Low | Low | Low | Low | Low | Good |
| Martinez et al. (2021) | Low | Low | Low | Low | Low | Low | Low | Good |
| Ribeiro et al. (2023) | Unclear | Low | Low | Low | Low | Low | Low | Good |

**Table S 2.** Risk of bias assessment using RoB 2 tool for included randomised controlled clinical trials in LACP

**Table S 3.** Summary of methods used for gut microbiota analysis across included studies.

| **Ref.** | **Quality and techniques of stool samples** | **Method** |
| --- | --- | --- |
| Crovesy et al. (2021) | The stool samples were collected at least one day before the consultation in flasks provided by the researchers, following the collection protocol. The transport was carried out in polystyrene, also provided by the researchers, with ice to preserve the sample. The samples were identified with the volunteer’s code and stored in a freezer at −80 °C until analysis | **DNA isolation:** using the QIAmp Fast DNA Stool Mini Kit  **RT-PCR of faecal microbiota species:** performed on StepOne Plus Real-Time PCR System (Life Technologies) to evaluate the phyla Firmicutes, Bacteroidetes, Actinobacteria, and Verrucomicrobia and the class γ-Proteobacteria.  **Quantification**: the relative abundance was normalised using the CT of total bacteria.  **Metabolite profile associated with changes in the gut microbiota analysis:** performed obtained using Bruker DRX 500 MHz equipment (Bruker Spectrospin, Karlsruhe, Germany).  One-and two-dimensional 1H-NMR spectra were processed using TopSpin 3.2 software (Bruker Biospin).  Metabolites were assigned using the Human Metabolome Database, and NMR data were processed using NMRLab. |
| Jamar et al. (2021) | The volunteers were instructed to collect from 20 to 30 g of fecal sample directly in a sterile flask according to the protocol of laboratory techniques for collection of faecal; otherwise, the material was kept at −20°C for up to 12 h until it was stored at − 80 °C. | **SCFA measurement:** Acetate, Propionate and butyrate were identified in a gas chromatography equipped with a flame ionisation detector (GC-FID) (Varian Star 3400CX, CA, USA) by comparing retention time in the ionic flame using a known standard of SCFA (Volatile Free Acid Mix, Sigma-Aldrich).  **DNA isolation:** using the QIAmp Fast DNA Stool Mini Kit (Qiagen, CA, USA).  **RT-PCR of faecal species:** using SYBR Green PCR Master Mix (Applied Biosystems) in a StepOne Real-Time PCR Systems (Applied Biosystems, Foster City, CA, USA) with each reaction mixture standardised for 20 ng DNA/μL.  The article provides information on primers and methods used for data analysis and statistics. |
| Martinez et al. (2021) | Not reported | **DNA isolation:** DNA isolation and amplification were extracted from stool samples, and the differential abundance of amplicon sequence variants (ASV) was monitored by high throughput 16S rRNA gene sequencing.  **Analysis:** in duplicate with two pipelines: QIIME plugin version of DEICODE.  Gut microbiota beta diversity analysis was done by compositional data analysis with DEICODE using a robust Aitchison PCA.  The article provides information on probes, primers, and methods used for data analysis and statistics. |
| Pena et al. (2014) | Not reported | DNAs from all fecal samples were processed by qPCR for detection of total bacteria and of the *Bifidobacterium*, *Lactobacillus*, *Enterococcus* and *Bacteroides* populations, using the Light Cycler Fast Start DNA Master SYBR Green I kit in a Light Cycler (Roche Diagnostics, Mannheim, Germany). |
| Ribeiro et al. (2023) | Subjects were instructed to collect fecal samples as close as possible to the analysis time and to keep the material under refrigeration (4 °C) until its delivery to the laboratory. Fecal samples were kept at −80 °C until the time of preparation for the analyses of intestinal microbiota, fecal pH, and SCFA. | **DNA isolation:** Total DNA from fecal samples was extracted using a standardised protocol. A series of steps involving the addition of buffers, lysis, precipitation, and centrifugation were performed to isolate DNA. The quality and quantity of the extracted DNA were assessed and stored at −20 °C.  **16S rRNA Bacterial Gene Sequencing:** Sequencing of the V4 hypervariable region of the 16S rRNA gene was outsourced to Argonne National Laboratory®. Library preparation was done using Reagent Kit v3 (Illumina), and sequencing occurred on the MiSeq platform. Demultiplexing, removal of adapters, barcodes, and primers, as well as quality control, were performed using the Qiime 2 program. The sequences were taxonomically classified using the SILVA 138 database. Raw sequences were deposited in the NCBI Sequence Read Archive under the access number PRJNA681193. |
